# 952. Impact of Dermal Template in Burn Centers: Retrospective Single-arm Cohort Analysis of National Burn Repository

**DOI:** 10.1093/jbcr/irag033.519

**Published:** 2026-04-06

**Authors:** Roselle E Crombie, Claire Witherel, Yi Arnold

**Affiliations:** Connecticut Burn Center/Yale New Haven Health, Bridgeport, CT; Integra LifeSciences, Metuchen, NJ; Integra LifeSciences, Ellicott City, MD

## Abstract

**Introduction:**

Burn injuries remain a prominent public health concern, with over 11 million burn injuries occurring worldwide each year with wide-ranging impacts on patient outcomes, healthcare systems, and resource utilization. The National Burn Repository (NBR) and the Burn Care Quality Platform (BCQP), both maintained by the American Burn Association, have captured comprehensive data that enables evaluation of cases of specific care algorithms, including product-specific data, along with subsequent clinical outcomes and other quality indicators across burn centers in the United States. This study describes a single-arm observational analysis of burn patients treated with a bilayer dermal regenerative template (DRT).

**Methods:**

A total of 1095 patients were identified as being treated with DRT (self-reported within the registry data) from the NBR and the BCQP datasets between 2021 and 2024. The key variables extracted for analysis included investigation of patient demographics, injury characteristics, treatment interventions, anatomical location, care outcomes, and discharge disposition.

**Results:**

Fifty-two percent of the patients suffered mixed second- and third-degree burns, whereas 25% had second degree and 22% had third degree burn injuries. Majority of patients with DRT had burns <10% total body surface area (TBSA) (48%) and 10-40% TBSA burn (32%); 16% of patients managed with DRT suffered large surface area burn (>40% TBSA). Patients who were treated with Integra mostly had burns originating from flame (53%) and scald (17%) injuries. The mean modified Baux score for all patients was 63.4 ± 33.3%. Over 75% of patients’ burns managed with DRT were in locations: hand, upper and lower arms, upper and lower legs, foot, and face. Complications included graft loss requiring reoperation (8%), superficial surgical site infection (3.5%), deep surgical site infection (2.4%), and sepsis (4%). Further, many of these patients also had comorbidities, including Diabetes mellitus (14%), obesity (9%), tobacco use (25.5%), and alcohol abuse (19%). Documented mortality rate was 5.5%, a majority, 52% were discharged home to self-care.

**Conclusions:**

These data demonstrated low rates of infection or graft loss compared to previously published rates (Gonzalez 2020 PRS). This data also provides anatomical location-level granularity to describe where DRT is most utilized for burn management. These findings underscore the importance of tailored care pathways and for specific areas of the body, and continued support for high-risk groups in optimizing burn outcomes.

**Applicability of Research to Practice:**

As technologies add to the cost and complexity of burn wound closure, understanding the impact on where best to utilize them must be studied.

**Funding for the study:**

Acquisition of the date was through and an educational grant.

